# Serum C-Reactive Protein and Interleukin-6 Levels as Biomarkers for Disease Severity and Clinical Outcomes in Patients with Idiopathic Granulomatous Mastitis

**DOI:** 10.3390/jcm10102077

**Published:** 2021-05-12

**Authors:** Yi-Min Huang, Chiao Lo, Chiao-Feng Cheng, Cheng-Hsun Lu, Song-Chou Hsieh, Ko-Jen Li

**Affiliations:** 1 Department of Internal Medicine, National Taiwan University Hospital Yunlin branch, Yunlin 640, Taiwan; ymwhuang@gmail.com (Y.-M.H.); chiaofengcheng@gmail.com (C.-F.C.); 2 Department of Surgery, National Taiwan University Hospital, Taipei 100, Taiwan; dtsurgz6@gmail.com; 3 Department of Internal Medicine, National Taiwan University Hospital, Taipei 100, Taiwan; b89401085@ntu.edu.tw (C.-H.L.); hsiehsc@ntu.edu.tw (S.-C.H.)

**Keywords:** C-reactive protein, disease severity, idiopathic granulomatous mastitis, interleukin-6, time to resolution

## Abstract

Idiopathic granulomatous mastitis (IGM) is a rare inflammatory breast disease mimicking breast cancer. Limited research has been conducted on the application of serum biomarkers. This study aims to investigate the association of serum biomarkers with disease severity in patients with IGM. From November 2011 to March 2020, medical records of patients with IGM were reviewed. Serum cytokine levels were measured in patients and healthy controls between July 2018 and March 2020. A total of 41 patients with histologically proven IGM were found. Serum interleukin (IL)-6 level was significantly higher in patients with IGM (*n* = 11) than healthy controls (*n* = 7). Serum IL-6 and C-reactive protein (CRP) levels were significantly higher in patients with severe disease than mild and moderate disease. Serum IL-6 (Spearman’s *ρ* = 0.855; *p* < 0.001) and CRP (Spearman’s *ρ* = 0.838; *p* = 0.001) levels were associated with time to resolution. A higher serum CRP level was associated with a longer time to resolution (B = 0.322; *p* < 0.001) in multiple linear regression analysis. Serum IL-6 and CRP levels can be used as biomarkers for the evaluation of disease severity in IGM. IL-6 may play a crucial role in the immunopathology of IGM.

## 1. Introduction

Idiopathic granulomatous mastitis (IGM) is a rare, chronic, and benign breast disease, which is characterized by non-caseating granulomatous inflammation. It was first reported by Kessler and Wolloch in 1972, and the clinical and radiographic features can simulate breast cancer [1]. The common clinical manifestations include tender breast mass, skin erythema, ulcer, fistula, and abscess formation [2]. Extramammary manifestations are found in 34% of the patients with IGM [3]. The typical extramammary manifestations include arthritis and erythema nodosum [4]. IGM is found mainly in the Asian, North African, and Hispanics [3]. Women of childbearing age with a history of parity and breastfeeding are predominantly affected, whereas nulliparous women, postmenopausal women, and men are seldom reported [5]. Although the etiology of IGM is not fully established, factors contributing to the development of IGM include hormones, autoimmunity, microorganisms, smoking, and α1-antitrypsin deficiency [6].

**Figure 1:**
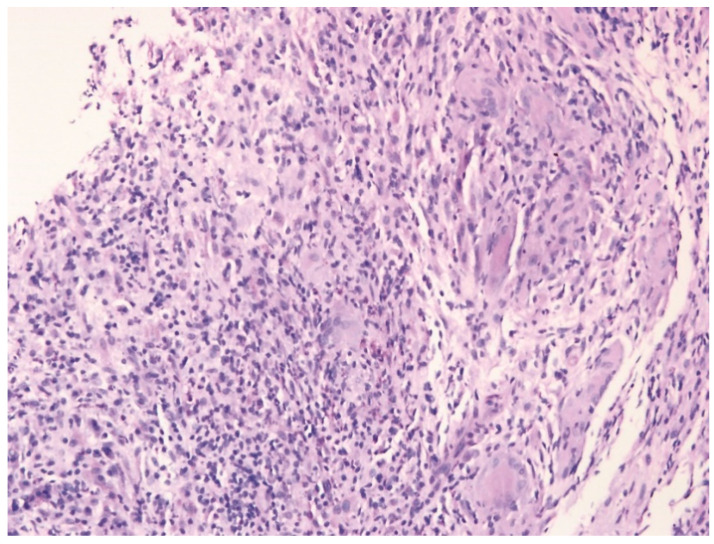
A histopathological examination showed vague non-caseating granulomatous inflammation with epithelioid histiocytes, lymphocytes, scattered neutrophils, and multinucleated giant cells in a patient with idiopathic granulomatous mastitis (H&E × 200).

The definite diagnosis of IGM requires histopathological examination and the exclusion of other etiologies [7]. The typical histopathological findings consist of well-formed or vague non-caseating granulomatous inflammation, epithelioid histiocytes, multinucleated giant cells, microabscess formation, and fibrosis (Figure 1). The infiltration of neutrophils, plasma cells, lymphocytes, and eosinophils can be found [8]. Based on the literature, surgical management or systemic glucocorticoid (GC) is widely considered as the initial treatment in IGM patients [3]. Moreover, topical GC, methotrexate (MTX), azathioprine, hydroxychloroquine, colchicine, and tumor necrosis factor (TNF) inhibitors have been reported to treat IGM [9,10,11,12].

The localized inflammatory response has been considered as one of the pathogenic factors of IGM. This hypothesis is supported by the lymphocytes infiltration in pathological sections, effectiveness of GC treatment, and presence of extramammary manifestations [4]. Recent studies had also found higher serum concentrations of interleukin (IL)-8, IL-10, IL-17, IL-22, IL-23, and IL-33 in IGM patients than healthy controls (HC) [13,14,15]. However, the association between clinical and immunological features of IGM remains unrevealed. This study aims to evaluate the association between serum biomarkers and clinical outcomes and investigate risk factors associated with treatment-refractory status.

## 2. Materials and Methods

### 2.1. Patients

From November 2011 to March 2020, medical records of patients with IGM at the National Taiwan University Hospital were retrospectively reviewed. Patients with histopathological evidence of IGM were included. Core-needle biopsy or excisional biopsy was performed for a definite diagnosis. Stains and cultures of pus or tissues were performed for bacteria, acid-fast organisms, and fungi. Patients with infection, malignancy, sarcoidosis, granulomatosis with polyangiitis, giant cell arteritis, and polyarteritis nodosa were excluded. Patients with previous pulmonary or extrapulmonary tuberculosis, contact history of patients infected with tuberculosis, pregnancy, silicone breast implants, and recent tetanus vaccination were also excluded.

### 2.2. Serum Assays

From July 2018 to March 2020, serum samples were measured in patients with IGM and HC. All samples had been stored at −80 °C until further analysis was performed. The concentrations of TNF-α, IL-1β, IL-2, IL-4, IL-6, IL-10, IL-12p70, IL-17A, IL-22, IL-23, and granulocyte–macrophage colony-stimulating factor (GM-CSF) were measured with the ProcartaPlex magnetic bead-based multiplex assay (Cat. No. EPX180-12165-901; eBioscience, Vienna, Austria). The manufacturer’s instructions were followed. The lower limit of detection for each cytokine was as follows: 0.4 pg/mL for TNF-α, 0.2 pg/mL for IL-1β, 0.8 pg/mL for IL-2, 1.5 pg/mL for IL-4, 0.4 pg/mL for IL-6, 0.1 pg/mL for IL-10, 0.04 pg/mL for IL-12p70, 0.1 pg/mL for IL-17A, 8.2 pg/mL for IL-22, 0.9 pg/mL for IL-23, and 1.2 pg/mL for GM-CSF. Values below the limit of detection were assumed to be one-half of the minimum detectable level for statistical and graphing purposes. All subjects were recruited according to a protocol approved by the Institution Review Board and Research Ethics Committees of National Taiwan University Hospital, Taipei, Taiwan (201705097RINB).

### 2.3. Treatments

Patients with IGM received initial management by experienced breast surgeons. Ultrasonography-guided fine needle aspiration was performed for breast abscess drainage. Surgical management was performed in patients with abscess formation or skin fistula after informed consent. Surgical management included incision and drainage, local excision, and partial mastectomy. As a result of cosmetic concerns, surgery was done in a minimally invasive manner. The benefits and risks of immunosuppressants were explained by experienced rheumatologists in a way that patients understood. Systemic GC was started in a shared decision-making approach. Dose tapering of systemic GC was initiated in the patients with significant treatment response after 2 weeks. Oral MTX was added to the patients who suffered from disease exacerbation during systemic GC dose reduction. MTX dosing was started at 7.5–10 mg weekly and increased to 15 mg weekly according to treatment response and physician’s judgments. Systemic GC was stopped gradually after the achievement of clinical resolution. Then, oral MTX was discontinued under sustained clinical resolution.

### 2.4. Data Collection

Information regarding patient demographics, clinical manifestations, obstetric history, serum C-reactive protein (CRP) level, treatment modalities, and clinical outcomes was obtained from medical records. Mild disease was defined as lesion size <2 cm, occasional mastalgia, and no abscess formation or skin ulcer; moderate disease was defined as lesion size less than 2−5 cm, small abscess formation with or without a skin fistula; severe disease was defined as lesion size >5 cm, refractory mastalgia, abscess formation requiring repeated drainage, and multiple skin ulcers and fistulas [16]. Laboratory data on the initial visit were analyzed. A clinical resolution was defined as a complete absence of symptoms [16,17,18,19,20]. Time to resolution was defined as the duration from initiation of medical or surgical management to the achievement of clinical resolution. Recurrence was defined as another episode of IGM after the achievement of clinical resolution of the initial episode for 3 months [16,17,19,20].

### 2.5. Statistical Analysis

The characteristics of the IGM patients with mild, moderate, and severe disease were compared with a Kruskal–Wallis test for continuous variables or a Chi-square test for categorical variables. When the Kruskal–Wallis test was significant, post hoc Dunn’s test was used to adjust for multiple comparisons to determine where differences existed. When the Chi-square test was significant, Fisher’s exact test and Bonferroni correction were performed for the pairwise comparisons between three groups. The serum concentrations of cytokines were compared with Mann–Whitney U tests. Plots were generated to illustrate the association between time to resolution and serum biomarkers, and Spearman’s correlation coefficients were computed. Before further analysis, each variable was examined for normal distribution by histogram and box plot. If a variable was not normally distributed, it was logarithmically transformed before the linear regression analysis. Time to resolution, body mass index, age of menarche, gestation, lesion size, and serum CRP level were transformed using the natural logarithm because of the skewed distribution. The association of time to resolution and clinical features was evaluated using simple linear regression analysis. Variables with *p* < 0.157 were included in the exploratory backward multiple linear regression model [21]. The association of recurrence and clinical features was investigated using univariate logistic regression analysis. All tests were two-tailed and *p* < 0.05 was considered statistically significant. Data analyses were conducted using SPSS software version 25.0 (SPSS Inc., Armonk, NY, USA).

## 3. Results

### 3.1. Characteristics According to Disease Severity

A total of 41 patients with histologically proven IGM were found from November 2011 to March 2020. Serum samples of 11 IGM patients and seven HC were collected. Clinical features and outcomes of IGM are presented according to disease severity (Table 1). Patients had a mean (SD) age of 35.9 (5.8) years and a median (IQR) lesion size of 4.0 (2.1–5.1) cm. The median (IQR) time of follow-up was 24.0 (14.5–42.5) months. Among the included patients, 20 (48.8%) presented with abscess formation, 29 (70.7%) presented with multiple lesions, 21 (51.2%) presented with skin ulcer or fistula, nine (22.0%) presented with bilateral lesions, and 11 (26.8%) presented with extramammary manifestations. The median (IQR) CRP level was 0.56 (0.14–2.89) mg/dL. Thirty-six (87.8%) patients were treated with systemic GC, 16 (39.0%) were treated with oral MTX, and 14 (34.1%) were treated with surgical management. All patients achieved clinical resolution during the study period. The median (IQR) time to resolution was 26.3 (12.4–56.0) weeks. Recurrence was found in nine (22.0%) patients. The median (IQR) time from resolution to recurrence was 24.0 (3.0–27.0) months. Lesion size, percentage of multiple lesions, percentage of skin ulcer or fistula, CRP, and time to resolution were significantly different between the IGM patients with mild, moderate, and severe disease (*p* < 0.001, *p* < 0.001, *p* = 0.008, *p* < 0.001, *p* = 0.002, respectively).

### 3.2. IL-6 and CRP as Biomarkers for Disease Severity

**Figure 2:**
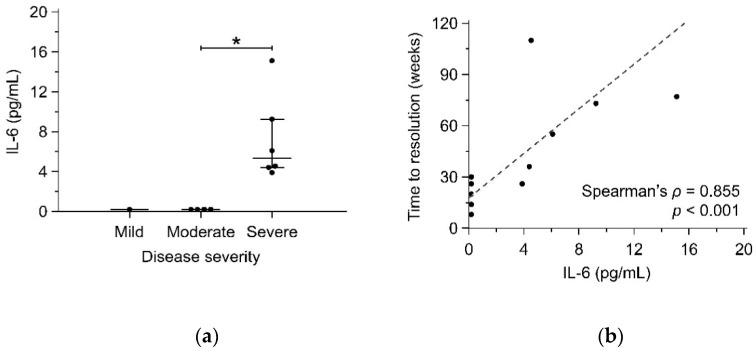

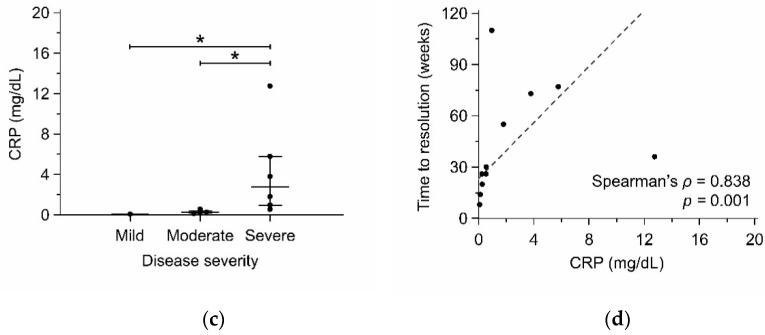
Serum IL-6 (**a**) and CRP (**c**) levels among IGM patients with mild, moderate, and severe disease were compared using the Kruskal–Wallis test and post hoc Dunn’s test. Spearman’s correlation coefficient was calculated to determine the correlation of serum IL-6 (**b**) and CRP (**d**) levels and time to resolution. Scatter diagrams and reduced major axis regression (dashed line) were displayed. * *p* < 0.05; CRP, C-reactive protein; IGM, idiopathic granulomatous mastitis; IL-6, interleukin-6.

The serum concentrations of TNF-α, IL-1β, IL-2, IL-6, IL-12p70, and IL-17A were significantly higher in patients with IGM than HC (Table 2). Among these cytokines, serum IL-6 level was significantly different between the IGM patients (*p* = 0.016) with mild (*n* = 1), moderate (*n* = 4), and severe disease (*n* = 6). Patients with severe disease had higher serum IL-6 levels than moderate disease (median: 5.32 vs. 0.00 pg/mL; adjusted *p* = 0.007) (Figure 2a). Moreover, serum IL-6 had a positive correlation with time to resolution (Spearman’s *ρ* = 0.855; *p* < 0.001) (Figure 2b). Among the patients with cytokine data, serum CRP level was positively correlated with serum IL-6 level (Spearman’s *ρ* = 0.830; *p* = 0.002). Serum CRP level was higher in the patients with severe disease (median: 2.80 mg/dL) than moderate disease (median: 0.26 mg/dL; adjusted *p* = 0.032) and mild disease (median: 0.08 mg/dL; adjusted *p* = 0.041) (Figure 2c). The CRP level also had a positive correlation with time to resolution (Spearman’s *ρ* = 0.838; *p* = 0.001) (Figure 2d).

### 3.3. Factors Associated with Time to Resolution

Factors associated with time to resolution in IGM patients are outlined in Table 3. In simple linear regression analysis, time to resolution had a positive association with skin ulcer or fistula (B = 1.480; *p* < 0.001), bilateral lesions (B = 1.325; *p* < 0.001), serum CRP level (B = 0.404; *p* < 0.001), and MTX use (B = 1.203; *p* < 0.001), and it had a negative association with breastfeeding (B = −1.188; *p* = 0.033) and smoking (B = −1.650; *p* = 0.032). In exploratory backward multiple linear regression analysis, time to resolution was independently associated with breastfeeding (B = −0.724; *p* = 0.040), bilateral lesions (B = 0.549; *p* = 0.047), serum CRP level (B = 0.322; *p* < 0.001), and MTX use (B = 0.707; *p* = 0.004) (Table 4).

### 3.4. Factors Associated with Recurrence

Factors associated with recurrence in IGM patients are outlined in Table 5. In univariate logistic regression analysis, age (odds ratio (OR) 1.18; 95% confidence interval (CI), 1.00−1.39; *p* = 0.049), skin ulcer or fistula (OR 11.69; 95% CI, 1.30−105.03; *p* = 0.028) and serum CRP level (OR 1.87; 95% CI, 1.14−3.08; *p* = 0.013) were factors associated with recurrence.

## 4. Discussion

This study revealed that serum CRP and IL-6 levels served as biomarkers for disease severity in patients with IGM. Moreover, both serum CRP and IL-6 levels were associated with time to resolution significantly. Among the clinical features, serum CRP level was one of the risk factors for a longer time to resolution and recurrence in our analysis. These findings implied that serum CRP and IL-6 levels were useful biomarkers for evaluating the severity of IGM. The association between serum IL-6 level and clinical outcomes expanded the understanding of the mechanism of inflammation in IGM. To our knowledge, only a few studies have examined the association between biomarkers and clinical outcomes, and this is the first study to reveal the role of IL-6 and CRP in IGM.

As the potential sources of IL-6, epithelioid histiocytes, multinucleated giant cells, and T cells were found in the pathological study of IGM [22]. Furthermore, the percentage of circulating effector T cells was higher in IGM patients than HC [23]. In addition, IL-6 together with IL-23 and transforming growth factor-β can induce T cell differentiation into T helper 17 (T_H_17) cells. T_H_17 cells will produce proinflammatory cytokines, IL-17, and IL-22 [24]. Our findings were compatible with the findings of a previous study which demonstrated that higher serum IL-17 concentration was found in IGM patients compared to HC [14]. The other study showed that the serum concentration of IL-22 and IL-23 was higher in IGM patients compared to HC; however, there was no statistically significant difference in serum IL-17 concentration [13]. The increased synthesis of CRP is primarily induced by IL-6, and to a lesser degree by IL-1β and TNF-α. A lower serum CRP level in patients with IGM compared to patients with breast cancer (median: 0.15 vs. 0.56 mg/dL; *p* < 0.001) was reported in a previous study [15]. However, we found a higher serum CRP level (median: 1.80 mg/dL) in patients with severe IGM. This discrepancy might be owing to the fact that the previous study did not take into account disease severity. The impact of breastfeeding on the prognosis of IGM is currently under investigation. A previous study indicated that the percentage of breastfeeding was higher in IGM patients with a longer time to resolution [25]. However, the treatment protocol was varied between the groups of different time to resolution. The same treatment protocol was applied to our cohort. After adjusting for other risk factors, we demonstrated that breastfeeding was associated with a shorter time to resolution. In contrast to the recent study, we did not find the association between the age of first live birth and time to resolution [26]. The mean age of first live birth of our cohort and the previous study were 31.1 and 21.6 years, respectively. This difference can lead to the inconsistency of association between age of first live birth and time to resolution. One possible explanation is that the influence of age of first live birth might be trivial due to longer exposure to hormone stimulation of mammary gland in our cohort.

An inflammatory biomarker, neutrophil-to-lymphocyte ratio (NLR), has been identified as a risk factor of recurrence in patients with IGM [27]. However, serum CRP level was not measured in the above study. The NLR represents the interaction between the innate and adaptive immune systems. Furthermore, the NLR has been known as a prognostic factor in cardiovascular disease and solid cancer [28,29]. Although the present study showed a numerical increase of NLR in IGM patients with recurrence, NLR and serum CRP level both were essential for evaluation of inflammation. Similar to our results, skin ulcer or fistula has been identified to be a risk factor for recurrence in the previous studies [30,31]. Therefore, we should be alert to the skin lesion of the breast in patients with IGM. Prompt treatment is required to prevent damage from persistent inflammation.

The results of the present study provided evidence for the association of dysregulated IL-6 production and disease severity in IGM. Our findings suggested a new link between the innate and adaptive immune systems in the complexity of IGM pathogenesis. In this study, elevated serum TNF-α, IL-1β, and IL-6 levels indicated the involvement of activated innate immunity in patients with IGM. TNF-α, IL-1β, and IL-6 are endogenous pyrogens and inducers of acute phase response [32]. On the other hand, elevated serum IL-2, IL-6, IL-12p70, and IL-17A levels supported the hypothesis of the aberrant T cell immunity in granuloma formation of IGM. IL-2 can induce T cell proliferation, and IL-12p70 and IL-6 can induce differentiation of CD4 T cells into the type 1 helper T (T_H_1) cells and T_H_17 cells, respectively [33]. T_H_1 and T_H_17 responses were both required in the development of granulomatous inflammation in tuberculosis and sarcoidosis [34,35]. However, the conflicting results of the T_H_17 response in IGM were observed in the aforementioned studies [13,14]. The findings of this study suggested that the T_H_17 response might be involved in the chronic inflammation in IGM. Further analyses are required to elucidate the relationship between T cell subsets and chronic inflammation in IGM.

The findings of this study suggested the association of serum CRP level and disease severity in IGM patients. Serum CRP measurement can be helpful not only in the assessment of disease severity but also in estimating time to resolution in patients with IGM. Patients with bilateral lesions were associated with time to resolution but not with disease severity. The disease severity might be underestimated in patients with bilateral lesions, who were assessed by a unilateral breast lesion. A careful assessment of the disease severity by physical examination and serum CRP level can provide useful information relating to the treatment of patients with bilateral lesions. Excessive cytokine production of IGM can be reduced by inhibition of inflammation. In a randomized controlled trial, numerical reduction of serum TNF-α, IL-1β, IL-2, and IL-6 was found in IGM patients after treated with Chuang Ling Ye, which is a traditional Chinese herbal medicine compound composed of rhubarb, safflower, *Abelmoschus manihot*, and *Terminalia chebula* [36]. With the understanding of cytokine dysregulation in IGM, anti-cytokine therapies may be a promising treatment option to eliminate the inflammation in IGM.

TNF inhibitors, including etanercept and adalimumab, have been successfully used to treat patients with IGM refractory to systemic GC treatment [9,12]. However, there is no study regarding the use of other TNF inhibitors (infliximab, golimumab, certolizumab pegol) in the treatment of IGM. The pathogenic role of dysregulated IL-6 in rheumatoid arthritis, juvenile idiopathic arthritis, Castleman disease, giant cell arteritis, and cytokine release syndrome has been supported by the efficacy of IL-6-targeted therapies [24]. Biologics targeting IL-6 signaling might also alleviate the localized inflammatory response of IGM. Granulomatous mastitis can be one of the manifestations of giant cell arteritis [7]. Tocilizumab was the first anti-IL-6 receptor monoclonal antibody to be approved to treat giant cell arteritis by the FDA and EMA [37]. Sirukumab, an anti-IL-6 monoclonal antibody, has shown the efficacy in decreasing disease flares of giant cell arteritis in a phase 3 trial with early termination [38]. The effectiveness of IL-6-targeted therapies in the treatment of IGM might be a pioneering research agenda in further pilot studies. Anakinra (IL-1 receptor antagonist), ustekinumab (anti-IL-12/23p40 monoclonal antibody), and secukinumab (anti-IL-17A monoclonal antibody) have demonstrated the effectiveness in the treatment of giant cell arteritis in case reports, case series, and non-controlled cohort studies [39,40,41,42,43]. Future studies are needed to investigate the potential therapeutic targets for anti-inflammation in IGM.

MTX was administered to IGM patients when exacerbation occurred during systemic GC dose reduction in our study. Consequently, MTX use was associated with a longer time to resolution. Combination therapy with systemic GC and MTX has been used in IGM patients with poor response to systemic GC alone [44]. Nevertheless, the effectiveness of MTX monotherapy in the treatment of IGM was demonstrated in recent studies [20,45]. The optimal therapeutic strategy for use of MTX in IGM patients remains to be delineated. Chronic low-grade inflammation is a hallmark of aging. In patients with breast cancer, serum CRP level is higher in the elderly compared to the younger group [46]. This study indicated that older age is associated with recurrence in IGM. The age-associated inflammatory state might participate in the complex mechanism of IGM recurrence. However, this finding needs to be confirmed in future studies.

The following limitations are noteworthy in the present study. First, the number of included patients was small because of the rarity of IGM. Only 11 patients and seven HC were biologically explored. A small number of HC can not reflect the true normal range of serum cytokine levels. Thus, the results of the comparison of serum cytokine levels between patients with IGM and HC should be interpreted with caution. Future studies with a larger sample size can help to provide robust evidence. However, we provided a novel perspective on the relationship between biomarkers and disease severity. This new evidence of cytokine dysregulation makes advances in the field of the immunopathology of IGM. Second, the serum cytokine levels of patients with IGM in the present study were lower than the studies mentioned above [13,14,36]. Furthermore, the serum cytokine levels were within the normal range in most of our patients. This discrepancy might be explained by the use of immunosuppressants in 87.8% of the patients with IGM in the present study. By contrast, the immunosuppressants were scarcely used in the previous studies [13,14,36]. The use of immunosuppressants should be taken into consideration while delineating the cytokine profiling in IGM. Third, the current classification of disease severity can not make an accurate assessment of disease activity [16]. A disease activity scoring system can improve risk stratification and monitoring of disease activity in patients with IGM. The association between serum CRP level and disease severity may help in the development of a practical scoring system. Last, the cytokine profiling of IGM can not explain the stimulation of serum IL-6 production. The production of IL-6 may be stimulated by IL-1 and Toll-like receptors [24]. The present study facilitates the exploration of upstream signaling pathways of serum IL-6 production in patients with IGM.

## 5. Conclusions

In conclusion, serum CRP and IL-6 levels served as biomarkers for disease severity and time to resolution in patients with IGM. The association between serum IL-6 level and clinical outcomes provides valuable insight into the mechanism of inflammation in IGM. Future studies need to investigate the interaction of IL-6 with other inflammatory biomarkers in the pathogenesis of IGM.

## Figures and Tables

**Table 1:** Clinical features, serum C-reactive protein level, treatment modalities, and outcomes of patients with idiopathic granulomatous mastitis according to disease severity.

Characteristics	All Patients (*n* = 41)		Mild (*n* = 5)		Moderate (*n* = 15)		Severe (*n* = 21)		*p*-Value	Adjusted *p*-Value		
										Mild vs. Moderate	Mild vs. Severe	Moderate vs. Severe
Age, mean (SD), years	35.9	(5.8)	34.8	(4.6)	35.1	(4.8)	36.8	(6.8)	0.635			
Body mass index, median (IQR), kg/m^2^	23.6	(20.6−26.1)	22.4	(20.2−27.0)	23.9	(21.1−26.2)	23.5	(20.1−27.1)	0.821			
Age of menarche, median (IQR), years	12.0	(12.0−13.0)	12.0	(11.0−14.0)	13.0	(12.0−13.0)	12.0	(12.0−13.0)	0.786			
Pregnancy	38	(92.7)	5	(100)	14	(93.3)	19	(90.5)	>0.900			
Gestation, median (IQR)	1.0	(1.0−2.0)	2.0	(1.5−2.5)	1.0	(1.0−2.0)	1.0	(1.0−2.0)	0.426			
Age of first pregnancy, mean (SD), years	31.1	(6.4)	31.0	(5.2)	30.9	(5.9)	31.2	(7.2)	>0.900			
Breastfeeding	37	(90.2)	5	(100)	14	(93.3)	18	(85.7)	0.782			
Smoking	2	(4.9)	1	(20.0)	0	(0.0)	1	(4.8)	0.360			
Diabetes mellitus	1	(2.4)	1	(20.0)	0	(0.0)	0	(0.0)	0.122			
Lesion size, median (IQR), cm	4.0	(2.1−5.1)	1.0	(0.6−1.6)	3.0	(2.1−4.8)	5.0	(3.8−6.0)	<0.001	0.031	<0.001	0.018
Abscess formation	20	(48.8)	0	(0.0)	8	(53.3)	12	(57.1)	0.087			
Multiple lesions	29	(70.7)	0	(0.0)	15	(100)	14	(66.7)	<0.001	<0.001	0.036	0.082
Skin ulcer or fistula	21	(51.2)	0	(0.0)	6	(40.0)	15	(71.4)	0.008	0.780	0.021	0.269
Symptom duration, median (IQR), weeks	8.0	(3.4−14.5)	1.3	(0.5−8.0)	7.6	(3.2−13.4)	10.0	(5.0−16.4)	0.111			
Bilateral lesions	9	(22.0)	0	(0.0)	2	(13.3)	7	(33.3)	0.221			
Extramammary manifestation	11	(26.8)	2	(40.0)	2	(13.3)	7	(33.3)	0.330			
Corynebacterium infection	10	(24.4)	0	(0.0)	3	(20.0)	7	(33.3)	0.305			
C-reactive protein, median (IQR), mg/dL	0.56	(0.14−2.89)	0.08	(0.05−0.23)	0.26	(0.13−0.83)	1.80	(0.75−5.14)	<0.001	0.571	0.002	0.007
Methotrexate use	16	(39.0)	1	(20.0)	4	(26.7)	11	(52.4)	0.251			
Surgery	14	(34.1)	0	(0.0)	7	(46.7)	7	(33.3)	0.162			
Time to resolution, median (IQR), weeks	26.3	(12.4−56.0)	8.0	(4.4−15.1)	20.3	(13.3−32.6)	54.7	(26.1−77.0)	0.002	0.328	0.004	0.061
Recurrence	9	(22.0)	0	(0.0)	1	(6.7)	8	(38.1)	0.051			

Data are presented as number (percentage) unless otherwise specified. SD, standard deviation; IQR, interquartile range.

**Table 2:** The comparison of serum cytokine levels of patients with idiopathic granulomatous mastitis and healthy controls.

Cytokines	IGM (*n* = 11)		HC (*n* = 7)		*p*-Value
	Median (IQR)		Median (IQR)		
TNF-α, pg/mL	11.66	(8.70−24.11)	7.21	(4.97−7.21)	<0.001
IL-1β, pg/mL	2.80	(1.59−3.52)	0.21	(N.D.−1.42)	<0.001
IL-2, pg/mL	13.77	(3.97−22.08)	2.63	(N.D.−6.97)	0.023
IL-4, pg/mL	N.D.		N.D.		N.A.
IL-6, pg/mL	3.89	(N.D.−6.09)	N.D.		0.023
IL-10, pg/mL	1.13	(0.55−1.59)	1.06	(0.81−1.99)	0.892
IL-12p70, pg/mL	6.39	(6.24−6.98)	6.10	(5.95−6.10)	0.016
IL-17A, pg/mL	1.13	(0.41−1.98)	N.D.		0.041
IL-22, pg/mL	N.D.	(N.D.−14.29)	N.D.	(N.D.−48.66)	0.885
IL-23, pg/mL	N.D.		N.D.		N.A.
GM-CSF, pg/mL	N.D.	(N.D.−3.52)	N.D.		0.118

IGM, idiopathic granulomatous mastitis; HC, healthy controls; IQR, interquartile range; TNF, tumor necrosis factor; IL, interleukin; N.D., not detectable; N.A., not applicable; GM-CSF, granulocyte–macrophage colony-stimulating factor.

**Table 3:** Factors associated with time to resolution in patients with idiopathic granulomatous mastitis.

Characteristics (*n* = 41)	Simple Linear Regression					
	Unstandardized B (SE)		95% CI			*p*-Value
Age, years	−0.009	(0.029)	−0.069	to	0.050	0.756
Body mass index, kg/m^2^	0.064	(0.887)	−1.731	to	1.858	>0.900
Age of menarche, years	0.501	(1.542)	−2.618	to	3.621	0.747
Pregnancy	−1.012	(0.630)	−2.285	to	0.262	0.116
Gestation	0.037	(0.332)	−0.636	to	0.710	>0.900
Age of first pregnancy, years	0.026	(0.028)	−0.030	to	0.082	0.345
Breastfeeding	−1.188	(0.538)	−2.276	to	−0.100	0.033
Smoking	−1.650	(0.740)	−3.147	to	−0.153	0.032
Diabetes mellitus	−1.175	(1.081)	−3.362	to	1.012	0.284
Lesion size, cm	0.435	(0.287)	−0.146	to	1.015	0.138
Abscess formation	0.417	(0.332)	−0.255	to	1.088	0.217
Multiple lesions	0.674	(0.356)	−0.047	to	1.394	0.066
Skin ulcer or fistula	1.480	(0.242)	0.991	to	1.969	<0.001
Symptom duration, weeks	0.083	(0.129)	−0.179	to	0.345	0.525
Bilateral lesions	1.325	(0.350)	0.618	to	2.032	<0.001
Extramammary manifestation	0.466	(0.375)	−0.292	to	1.224	0.221
Corynebacterium infection	0.666	(0.380)	−0.102	to	1.434	0.087
C-reactive protein, mg/dL	0.404	(0.071)	0.260	to	0.548	<0.001
Methotrexate use	1.203	(0.289)	0.619	to	1.787	<0.001
Surgery	−0.131	(0.356)	−0.852	to	0.590	0.715
Recurrence	0.784	(0.389)	−0.003	to	1.572	0.051

SE, standard error; CI, confidence interval.

**Table 4:** Backward multiple linear regression model for prediction of time to resolution in patients with idiopathic granulomatous mastitis.

Characteristics (*n* = 41)	Multiple Linear Regression (Backward)					
	Unstandardized B (SE)		95% CI			*p*-Value
Breastfeeding	−0.724	(0.339)	−1.414	to	−0.034	0.040
Smoking	−1.001	(0.489)	−1.898	to	0.091	0.074
Bilateral lesions	0.549	(0.266)	0.008	to	1.089	0.047
C-reactive protein, mg/dL	0.322	(0.086)	0.148	to	0.497	<0.001
Methotrexate use	0.707	(0.230)	0.239	to	1.176	0.004
Recurrence	−0.612	(0.322)	−1.266	to	0.042	0.066

SE, standard error; CI, confidence interval.

**Table 5:** Factors associated with recurrence in patients with idiopathic granulomatous mastitis.

Characteristics (*n* = 41)	Univariate Logistic Regression				
	Odds Ratio	95% CI			*p*-Value
Age, years	1.179	1.000	to	1.390	0.049
Body mass index, kg/m^2^	0.969	0.827	to	1.135	0.697
Age of menarche, years	1.113	0.604	to	2.052	0.731
Pregnancy	0.533	0.043	to	6.655	0.625
Gestation	1.817	0.873	to	3.783	0.110
Age of first pregnancy, years	1.027	0.904	to	1.166	0.687
Breastfeeding	0.828	0.075	to	9.074	0.877
Smoking	N.A.				
Diabetes mellitus	N.A.				
Lesion size, cm	1.165	0.737	to	1.843	0.514
Abscess formation	2.571	0.545	to	12.139	0.233
Multiple lesions	1.591	0.279	to	9.066	0.601
Skin ulcer or fistula	11.692	1.302	to	105.028	0.028
Symptom duration, weeks	1.016	0.975	to	1.059	0.456
Bilateral lesions	4.320	0.851	to	21.929	0.078
Extramammary manifestation	2.857	0.601	to	13.586	0.187
Corynebacterium infection	0.857	0.147	to	4.999	0.864
C-reactive protein, mg/dL	1.874	1.139	to	3.084	0.013
Methotrexate use	4.400	0.911	to	21.248	0.065
Surgery	0.955	0.199	to	4.571	>0.900
Time to resolution, weeks	1.015	0.993	to	1.038	0.171

CI, confidence interval; N.A., not applicable.

## Data Availability

All data presented in this study are available on demand from the corresponding author.
